# Pulmonary Embolism (PE) to Chronic Thromboembolic Pulmonary Disease (CTEPD): Findings from a Survey of UK Physicians

**DOI:** 10.3390/arm92010007

**Published:** 2024-01-09

**Authors:** Joanna Pepke-Zaba, Luke Howard, David G. Kiely, Shruti Sweeney, Martin Johnson

**Affiliations:** 1 Pulmonary Vascular Diseases Unit, National Pulmonary Hypertension Service, Royal Papworth Hospital, Cambridge CB2 0AY, UK; joanna.pepke-zaba@nhs.net; 2 National Pulmonary Hypertension Service, Hammersmith Hospital, London W12 0HS, UK; lukehoward@nhs.net; 3 Sheffield Pulmonary Vascular Disease Unit, Royal Hallamshire Hospital, Sheffield Teaching Hospitals NHS Foundation Trust, Sheffield S10 2JF, UK; david.kiely1@nhs.net; 4 Department of Infection, Immunity and Cardiovascular Disease, University of Sheffield, NIHR Biomedical Research Centre, Sheffield S10 2RX, UK; 5 Medical Affairs Department, Janssen-Cilag Ltd., High Wycombe HP12 4EG, UK; ssween12@its.jnj.com; 6 Scottish Pulmonary Vascular Unit, Golden Jubilee National Hospital, Glasgow G81 4DY, UK

**Keywords:** pulmonary embolism, chronic thromboembolic pulmonary disease, chronic thromboembolic pulmonary hypertension, pulmonary hypertension

## Abstract

**Highlights:**

**What are the main findings?**

- Considerable variability was reported in the follow-up of patients presenting with acute PE and in the awareness and investigation of suspected CTEPD;
- Despite most participants having local guidelines for PE management, less than two-thirds reported that a dedicated PE follow-up clinic was available.

**What is the implication of the main findings?**

- These data suggest that a national audit should be performed to gain an understanding of the barriers to the timely detection of CTEPD among UK physicians.

**Abstract:**

Chronic thromboembolic pulmonary disease (CTEPD) is a complication of pulmonary embolism (PE). We conducted an online survey of UK PE-treating physicians to understand practices in the follow-up of PE and awareness of CTEPD. The physicians surveyed (*N* = 175) included 50 each from cardiology, respiratory and internal medicine, plus 25 haematologists. Most (89%) participants had local guidelines for PE management, and 65% reported a PE follow-up clinic, of which 69% were joint clinics. Almost half (47%) had a protocol for the investigation of CTEPD. According to participants, 129 (74%) routinely consider a diagnosis of CTEPD and 97 (55%) routinely investigate for CTEPD, with 76% of those 97 participants investigating in patients who are symptomatic at 3 months and 22% investigating in all patients. This survey demonstrated variability in the follow-up of PE and the awareness of CTEPD and its investigation. The findings support the conduct of a national audit to understand the barriers to the timely detection of CTEPD.

## 1. Introduction

Chronic thromboembolic pulmonary disease (CTEPD) is a complication of acute pulmonary embolism (PE) [1,2]. More research has been conducted on CTEPD with pulmonary hypertension (PH) (chronic thromboembolic pulmonary hypertension, CTEPH), while the natural history of CTEPD without PH and how it relates to CTEPH are unclear [3]. The cumulative 2-year incidence of CTEPH was recently estimated to be 2.3% in acute PE patients in the prospective, multicentre and observational FOCUS study [4], and the presence of PH confers a poorer prognosis. Left untreated, the historical 5-year survival rate for severe (mean pulmonary artery pressure: >50 mmHg) CTEPH is 10% [5].

The modern treatment for CTEPH is multimodal: pulmonary endarterectomy (PEA), balloon pulmonary angioplasty and medical treatment for PH may be used singly or in combination [2]. For those with a surgically accessible disease, PEA offers the best prospect of a cure [1]. Using an international, prospective registry of CTEPH patients, it was found that the 3-year survival was 89% in operated patients (*N* = 404) versus 70% in non-operated patients (*N* = 275) [6], and a UK study of 550 CTEPH patients found that the 5-year survival was superior in patients who underwent PEA (83%) versus patients who either declined surgery (53%) or were ineligible for surgery (59%) [7]. According to the treatment guidelines, the possibility of CTEPH should be considered in all symptomatic patients with breathlessness after 3 months of therapeutic anticoagulation [2,8]. Diagnostic algorithms for CTEPH recommend an echocardiographic assessment of the probability of PH, assessment of lung perfusion (e.g., using ventilation/perfusion (V/Q) lung scintigraphy) and cardiopulmonary exercise testing (CPET) [1,8,9,10]. However, CTEPH awareness is low among PE-treating physicians [11,12,13], and CTEPH often goes undiagnosed for over a year [14], negatively impacting patient prognosis, with higher pulmonary artery pressures and an increased risk of death [15].

We aimed to understand the level of awareness of CTEPD (with or without PH) among UK physicians and the current practices in the follow-up of patients presenting to hospital with PE. The key takeaways of this study are shown in Box 1.

Box 1 **Key takeaways from the CONNECT survey of UK PE-treating physicians.**

- CTPA is most often used as the first-line of investigation for CTEPD fol-lowing PE despite cardiac echocardiography and V/Q scintigraphy being recommended;
- CTEPH was ranked as the most common cause for persistent breathless-ness post-PE despite it being relatively rare compared to other issues such as deconditioning. This may lead to onward referrals without the appro-priate investigations being conducted;
- Haematologists with an interest in thrombosis should be involved in the PE follow-up pathway;
- Protocols for PE follow-up should highlight the need for a retrospective review of the CTPA from the initial PE assessment;
- Hospital protocols should ensure smooth integration of their PE care pathways and ensure that physicians are aware of their nearest specialist PH centre.

## 2. Materials and Methods

This was a cross-sectional online survey of PE-treating pulmonologists, cardiologists, haematologists and internal medicine specialists (other hospital departments were excluded) in the UK (other countries excluded), performed between 2 December 2021 and 24 January 2022. Eligible physicians were either Specialty Registrars or Consultants (other job titles excluded); had been working in their specialty for 3–40 years; were based in a district general hospital, teaching hospital, PH centre or PH shared care centre in the UK (other primary work settings excluded); and personally managed ≥10 PE cases in a typical year (those managing fewer than 10 cases were excluded). Participants were advised that their personal information would not be passed to a third party without their permission, that personal data would be destroyed after 2 years and that they had the right to withhold information or withdraw at any time. Participants were also informed that the research was treated in accordance with the European Society for Opinion and Marketing Research (ESOMAR) and the Market Research Society (MRS)’s Code of Conduct, that all data would be anonymised and that they would not be asked to provide data on an individual patient. Participants were advised that the survey findings were intended to be shared externally, including publication in a relevant peer-reviewed journal. Finally, participants were informed that it would be a requirement that any possible adverse events, product complaints or special reporting situations related to the study sponsor’s products were passed on (anonymously), even if already reported via the Medicines and Healthcare products Regulatory Agency’s (MHRA) ‘Yellow Card’ system, and they were asked if they would still like to proceed.

The survey was conducted by Sermo using the Decipher survey platform (FocusVision) and developed by the authors. It consisted of seven screening questions: two background questions, 10 questions on PE management and 14 questions on experience in evaluating suspected CTEPD (Supplementary Materials). The questions used nomenclature following the European Respiratory Society statement on CTEPH, including the term ‘CTEPD’ rather than ‘chronic thromboembolic disease’ [16]. The questionnaire took approximately 20 min to complete, and respondents were remunerated for their participation in accordance with fair market value rates.

### 2.1. Data Analysis

No formal statistical testing was performed; the analyses were purely descriptive.

### 2.2. Ethical Statement

This research complied with the UK data protection law (GDPR) and with the British Healthcare Business Intelligence Association’s (BHBIA) Legal & Ethical Guidelines, along with the European Pharmaceutical Market Research Association’s (EPHMRA) guidelines.

## 3. Results

### 3.1. Participants

The target of 175 physicians completing the questionnaire was reached: 50 each from respiratory medicine, cardiology and internal medicine, plus 25 from haematology. The response rate of the individuals approached for this study was 11%. The respondents were mainly consultants (88%) based in district general or teaching hospitals (82%) (Table 1).

### 3.2. General Management of PE in the Centres in Which the Respondents Practice

Eighty-nine percent reported that their centre had local guidelines on PE management (Table S1). The percentage of physicians reporting that their centre had a PE response team (PERT) ranged from 14% (internal medicine) to 54% (respiratory medicine) (Table S1).

The imaging modalities that physicians had access to are summarised in Table S2. All but two physicians reported access to computed tomography (CT) imaging (two selected “not sure which, if any, [CT] modalities are available”), while—of the nuclear medicine modalities—77% had access to V/Q scanning, 10% Q scan only and 22% single-photon emission computed tomography (SPECT). For the magnetic resonance imaging (MRI) modalities, 51% of physicians reported access to pulmonary angiography and 30% to lung perfusion mapping. Thirty-nine (22%) physicians reported access to conventional pulmonary angiography or digital subtraction angiography.

**Figure 1:**
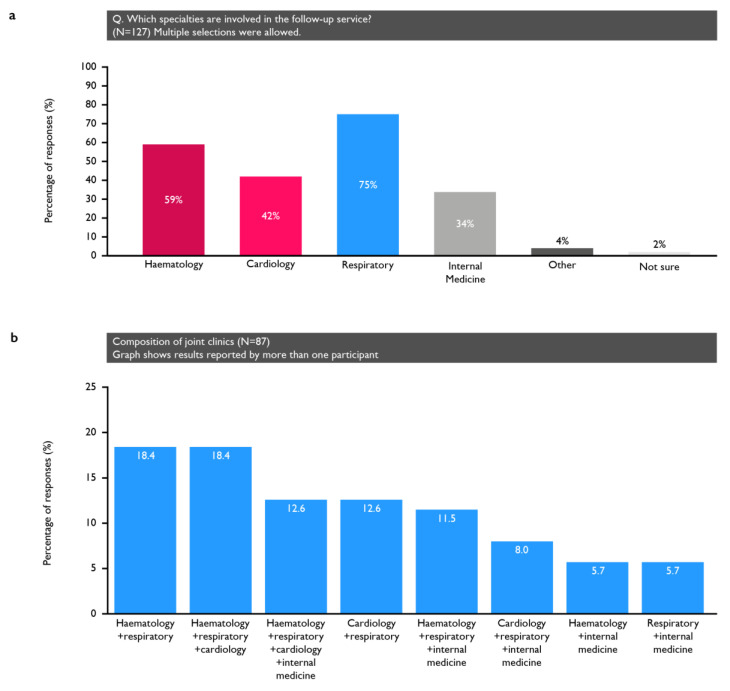
(**a**) Specialties involved in those centres that had dedicated clinics; (**b**) the composition of the clinics that involved multiple specialties.

Fifty-one percent of respondents reported a dedicated clinic for the follow-up of all PE patients (including low-risk, outpatient-managed patients) and 14% ran a clinic for hospital-admitted patients only (Table S1). The specialties involved in the follow-up service are shown in Figure 1. Of the 127 participants reporting a dedicated clinic, 87 (68.5%) had joint clinics, most commonly comprising either pulmonologists and haematologists or pulmonologists, haematologists and cardiologists (with 16 participants reporting each; 18%). Additionally, 65% of physicians reported that their clinic had a specialist nurse (12% were not sure).

**Figure 2:**
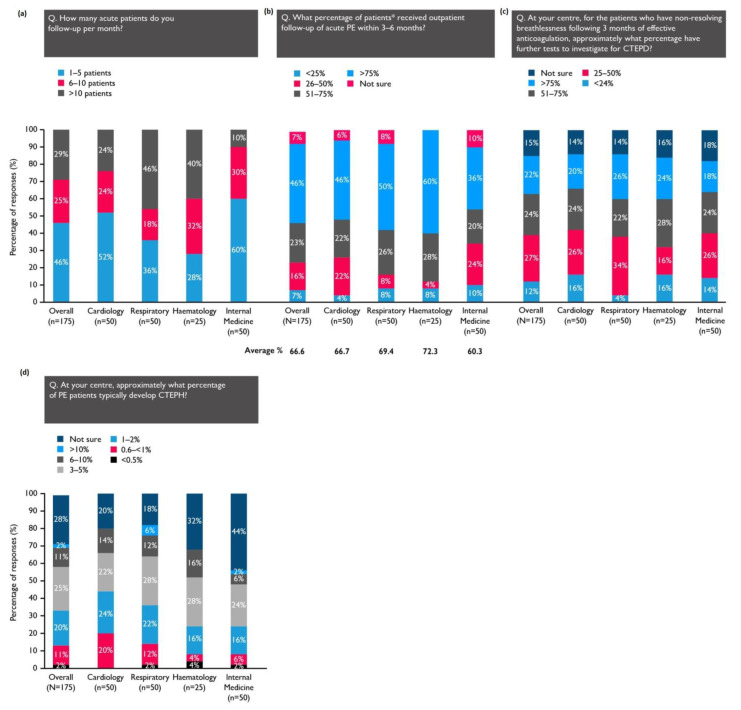
* Of those managed as outpatients at the physician’s centre. (**a**) The number of PE patients who are seen by the respondents; (**b**) the percentage of those that receive a follow-up within the recommended timeframe; (**c**) the percentage of patients with persistent dyspnoea after 3 months of therapeutic anticoagulation who are investigated for CTEPD; (**d**) the percentage of patients who typically develop CTEPH, according to the respondents.

On average, the physicians’ centres saw 24 acute PE patients each month, with two-thirds receiving outpatient follow-up within 3–6 months (Figure 2). The patients treated by haematologists and pulmonologists were the most likely to receive a follow-up during this time frame (Figure 2b).

The local guidelines for the follow-up of PE patients were followed by 63% of physicians, while 62% followed British Thoracic Society (BTS) guidance, 48% followed National Institute of Health and Care Excellence guidelines, 35% followed European Society of Cardiology (ESC) guidelines and 7% the American College of Chest Physicians guidelines.

### 3.3. Experience in Evaluating Suspected CTEPD

A diagnosis of CTEPD was routinely considered by 74% of the respondents following acute PE (84% cardiology, 88% respiratory, 72% haematology and 50% internal medicine) and routinely investigated for by 55% of the respondents (Table 2). Notably, fewer haematologists would investigate patients with thrombophilias for CTEPD (8%) than other specialties (31–40%).

**Figure 3:**
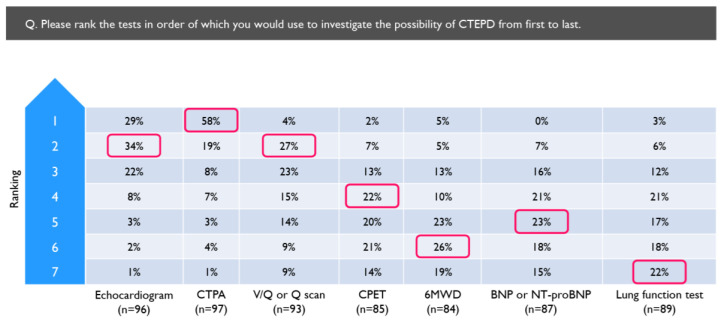
Tests ranked in the order they would be used to investigate for CTEPD by physicians who routinely investigate for CTEPD following PE (*N* = 97). Red boxes indicate the most common ranking allocated to each investigation. 6MWD = 6 min walking distance; BNP = brain natriuretic peptide; CPET = cardiopulmonary exercise test; CTPA = computed tomography pulmonary angiogram; NT-proBNP = N-terminal pro-brain natriuretic peptide; Q = perfusion; V/Q = ventilation/perfusion.

Echocardiogram was the test most often used to investigate for CTEPD, followed by computed tomography pulmonary angiogram (CTPA) (Table 2), while CPET was used the least often, although over half of the physicians reported using it. When asked to rank the tests in the order of use, physicians most often selected CTPA as the first-line test (58%), followed by echocardiogram (29%), 6-minute walking distance (6MWD) (5%) and V/Q scan (4%) (Figure 3).

All respondents were asked to rank the following from the most to least likely cause of persistent dyspnoea in PE patients: CTEPD, CTEPH, lung disease, left heart disease, deconditioning and dysfunctional breathing/anxiety. The condition most often ranked first was CTEPH (32%), followed by lung disease (21%) and CTEPD (20%) (Figure S1).

Most respondents (80%) reported knowing where their regional PH centre is located (Tables S3 and S4). The most common stage at which patients were typically referred to the PH service was at echocardiographic signs of PH or right heart dilatation, for most specialists (Table S5). Notably, 42% of physicians, overall, said they would refer patients who were still symptomatic after 3 months of anticoagulation without conducting any further tests (42%), and 38% reported they would refer breathless patients even if tests were normal.

Overall, 31% of respondents always retrospectively review the CTPA from the initial PE assessment for signs of CTEPH, 42% sometimes, 19% occasionally and 9% never do, with pulmonologists being the most likely (84% always/sometimes) to do so and internal medicine specialists the least likely (40% occasionally/never). Twenty-six percent of respondents always or sometimes used a risk score calculator to estimate the CTEPH probability; however, 43% were unaware of such a tool (Figure S2). Similarly, 32% of physicians always or sometimes used a dyspnoea score, and 37% were unaware of such a score. Most (82%) physicians did not use quality-of-life (QoL) questionnaires.

When asked to consider the patients at their centre with nonresolving breathlessness after 3 months of effective anticoagulation, 46% of physicians estimated that more than half of these patients underwent further tests to investigate for CTEPD (Figure 2c). Overall, 28% of respondents were not sure how many PE patients at their centre typically develop CTEPH; 57% believed the incidence to be 0.6–5% (Figure 2d).

## 4. Discussion

Most participants reported that their centre had local guidelines on PE management, but only half had a protocol for the investigation of CTEPD. Only 65% of physicians reported patients being followed up in a dedicated PE clinic, and only 22% reported that nonresolving breathlessness was investigated for CTEPD in >75% of cases. There was large variation in the follow-up of acute PE patients and in the awareness and approaches to the investigation of suspected CTEPD. The key takeaways are summarised in Box 1.

### 4.1. PE Management

It is encouraging that 89% of the respondents considered there to be local guidelines for PE follow-up. This is consistent with the results of the first BTS audit of the outpatient management of PE (audit period: September and October 2021), which demonstrated that 80% of participating UK centres (*n* = 108) had a formalised outpatient PE pathway [17]. Thirty-eight percent of respondents reported that their centre had a PERT; however, of these respondents, 47% were based in London and the term ‘PERT’ was not defined in our survey. Therefore, further study is needed to confirm how many centres have a PERT; studies should also investigate how these PERTs operate and which treatment options they have available.

### 4.2. Use of Imaging Modalities

All except two respondents (who were not sure) reported access to CT scans, and most had access to some form of nuclear imaging. Similarly, the BTS audit of outpatient PE management found that 92% of UK centres had 7-day access to CTPA, while 10% did not have access to nuclear imaging for patients unable to undergo CTPA [17].

The ESC/European Respiratory Society (ERS) guidelines on PE and PH recommend the V/Q scan (or an alternative perfusion imaging modality) for first-line imaging in suspected CTEPH [1,2,8]; however, 10% of respondents only had access to Q scan imaging, although the added value of ventilation imaging when other complementary imaging modalities are available is limited. There has been a gradual move toward the use of SPECT, as it is superior to planar V/Q imaging (Table S6) [16,18]. In our study, 22% of survey participants had access to SPECT. The survey results also show that CTPA and echocardiography were preferred over V/Q scanning as the first test to investigate for CTEPD (used first by 58%, 29% and 4% of physicians, respectively). The reasons for this were not captured, but it could be due to a greater availability of CT and echocardiography versus V/Q scans or the advantage of being able to look at the heart and lungs with CT imaging (Table S6) [18]. Test suitability may also vary among patients; for example, a V/Q scan may be preferred for young female patients for whom exposure to CTPA radiation is a concern or in cases where the interpretation of the CTPA findings is difficult, such as for patients with a higher cardiac output or where there may be concerns regarding the ability of the patient to cooperate with the breath-holding requirements during the CT imaging. It should also be noted that 80% of physicians always or sometimes used lung function tests (80%) to investigate the possibility of CTEPD, despite these tests not being as discriminatory as the imaging tools. It is important to conduct appropriate tests, and this finding suggests there is room for improvement.

Access to other imaging modalities was higher than expected. This survey could have been completed by more than one respondent from the same centre, thereby falsely inflating these percentages; however, the distribution of respondents across the regions and specialties suggests that the level of this type of ‘duplication’ is not excessive.

### 4.3. Other Tools to Support Post-PE Follow-Up

The use of CTEPH risk scores varied across respondents; however, only one such tool has been validated [19]. The In Shape II validation study of 424 patients found that the tool detected most (10 of 13) CTEPH patients within 4 months following PE [20]. However, when applied to 2256 PE patients in the Computerized Registry of Patients with Venous Thromboembolism (RIETE), its sensitivity was found to be too low (27.3%) to rule out CTEPH [21]. CTEPH risk score calculators may prove particularly useful when resources are limited, but they have some disadvantages. Because they are focused on distinguishing PH from non-PH, they do not help diagnose patients without PH. Also, completing the risk score before deciding on the next steps can require more visits than simply booking a test and the subsequent follow-up appointment at the same visit.

In our study, 18% of physicians reported asking their patients to complete QoL questionnaires. The only validated PE-specific QoL tool [22] was developed before quality standards for such instruments were devised and does not fully adhere to the guidelines [23]. The low use reported in this survey is consistent with the authors’ observation that QoL questionnaires tend to be used in clinical trials rather than in clinical practice.

### 4.4. Aspects of Post-PE Follow-Up Needing Improvement

The BTS audit of outpatient PE management demonstrated that 87% of UK centres performed follow-up at 3 months, with this occurring in a dedicated PE clinic in 36% of the centres [17]. In comparison, we found that 65% of the respondents’ centres had a dedicated PE clinic, but only 46% of the physicians reported that more than three-quarters of patients were offered a 3–6-month follow-up. A retrospective study of adults discharged from a UK teaching hospital with a diagnosis of acute PE in the first half of 2019 found that 31% of intermediate–high-risk and 37% of intermediate–low-risk patients were not followed up [24]. The authors hypothesised that dedicated PE clinics with multidisciplinary team meetings could streamline post-PE follow-up and improve outcomes [24].

The reasons for this gap in care are unknown; however, our finding that 74% of respondents routinely consider a diagnosis of CTEPD would suggest that lack of awareness is not predominant. Indeed, the high percentage of respondents rating CTEPD and CTEPH among the most common reasons for persistent symptoms following PE indicates that physicians are mindful of these conditions. Importantly, however, this finding reflects an inaccurate perception of the prevalence of CTEPD and CTEPH. Persistent dyspnoea and/or poor physical performance are relatively common several months after acute PE, but most cases are attributable to muscle deconditioning (ranked as the most likely reason by only 18% of survey respondents) [1,2]. This finding could reflect that physicians’ answers are biased because they are aware the survey is related to CTEPD. The availability of the CT and V/Q scanning modalities reported by the respondents (99% and 90%, respectively) indicates this is not a significant barrier to providing care.

The CLARITY survey found that the most frequent barriers affecting the detection of CTEPH after PE reported by respondents (353 physicians from Europe, Asia–Pacific and the Americas) were low disease awareness among nonexperts (77%), lack of structured follow-up after acute PE (56%), nonspecific presentation of disease (43%), incomplete understanding of the natural history of disease (39%) and lack of clinical guidelines to screen for possible CTEPH (73, 23%) [25]. Moreover, low adherence to guidelines was reported as a barrier to CTEPH diagnosis by approximately one-third of respondents [26]. The variability in the physicians’ practices, as shown in this survey, could support this finding that more comprehensive guidelines on PE follow-up are needed.

The survey pinpointed a specific practice that could be improved: 28% of physicians overall and 40% of internal medicine specialists occasionally or never review the CTPA from the initial PE assessment for signs of CTEPH. The In Shape III study showed that experts’ overall judgement on whether CTPAs at acute PE presentation showed signs of CTEPH achieved a sensitivity of 72% and a specificity of 94% [27]. A subsequent study demonstrated that nonexpert readers could also accurately detect CTEPH based on specific radiological signs according to a scoring form [28]. Therefore, routinely performing closer examination of initial CTPAs could improve detection of CTEPH.

### 4.5. Multidisciplinary Follow-Up of PE

In our survey, two-thirds of dedicated follow-up clinics were jointly run by more than one specialty. The benefits of such multidisciplinary clinics have been described previously [24]. A clear pathway for the follow-up of patients presenting with PE who are already under other specialties and have risk factors for PE (e.g., oncology) will be important [29,30]. In our study, the lower percentage of haematologists than other specialties routinely investigating for CTEPD in patients with thrombophilia likely reflects an understanding that investigations in these cases do not change disease management in many cases and suggests that the involvement of haematologists in clinic reviews could potentially avoid unnecessary investigations. Internal medicine specialists reported lower rates of follow-up with PE patients and consideration of CTEPD as a diagnosis. One of the BTS quality standards for the outpatient management of PE is that follow-up should be performed by a senior clinician with special interest in PE, as part of a formal pathway [31].

### 4.6. Referral to PH Centre

Most respondents were aware of the location of their regional PH centre. There are seven adult specialist PH centres in the UK; three are in London and the others are in Newcastle, Glasgow, Cambridge and Sheffield. Hospital protocols should ensure smooth integration of their PE care pathways.

According to the ESC and ERS guidelines for the management of PE and PH, symptomatic patients with persistent mismatched perfusion defects 3 months following acute PE should be referred to a specialist centre after consideration of the results of echocardiography, brain natriuretic peptide (BNP)/N-terminal pro-brain natriuretic peptide (NT-proBNP) and/or CPET investigations [2,8]. However, 42% of respondents in our survey reported that they typically referred (some) breathless patients without performing further tests, and 38% (sometimes) referred patients even if the subsequent test results were normal. A referral before conducting any further tests may be appropriate where there is a high index of suspicion based on original imaging, echocardiographic findings and medical history; however, in the majority of cases, patients would undergo further tests beforehand, as per guideline recommendations [1,2]. In cases in which patients are referred even if tests are normal, access to V/Q scintigraphy may prevent unnecessary referrals or support referrals where appropriate.

### 4.7. Strengths and Limitations

The main strength of this study is that it included 175 physicians from multiple specialties and different regions of the UK. There are methodological limitations associated with performing surveys, including recall bias and the potential for the survey sample to not be genuinely representative of the study population. In this case, the response rate was low (in keeping with studies of this type) and may have led to bias, the renumeration of participants for their time spent on survey might have influenced the survey population and the potential for more than one physician from a centre to have responded could have skewed the results. In addition, no formal sample size calculation was performed. These limitations could impact the generalisability of the findings. It should be noted that this survey was performed without being pilot-tested and that the inability to capture respondents’ reasons behind their answers may hinder a full understanding of the findings.

## 5. Conclusions

Our survey results demonstrated a wide variation in clinical practice in the follow-up of patients presenting with acute PE and in the awareness and approaches to the investigation of suspected CTEPD. This could indicate that more comprehensive and structured guidelines for PE follow-up are needed. These data support the conduct of a national audit to improve our understanding of the barriers to the timely detection of CTEPD.

## Figures and Tables

**Table 1:** Respondent demographics and characteristics.

	Cardiology (*n* = 50)	Respiratory Medicine (*n* = 50)	Haematology (*n* = 25)	Internal Medicine (*n* = 50)	Overall (*N* = 175)
**Title, *n* (%)**					
Specialty Registrar	10 (20)	6 (12)	0	5 (10)	21 (12)
Consultant	40 (80)	44 (88)	25 (100)	45 (90)	154 (88)
**Mean duration of experience in the specialty, years**	16.0	15.1	14.7	16.7	15.7
**Mean number of patients with PE seen in a year**	53.5	74.6	55.0	52.8	59.5
**Primary work setting, *n* (%)**					
District general hospital/teaching hospital	(66)	(82)	(92)	(94)	(82)
PH shared care centre	(12)	(14)	(4)	(4)	(9)
PH centre	(22)	(4)	(4)	(2)	(9)
**Hospital setting, n (%)**					
Public	46 (92)	47 (94)	19 (76)	45 (90)	157 (90)
Private	0	0	3 (12)	1 (2)	4 (2)
Both	4 (8)	3 (6)	3 (12)	4 (8)	14 (8)
**UK region of practice (%)**					
Northeast England	2	4	4	6	4
Northwest England	12	16	8	8	11
East Midlands	8	8	12	12	10
West Midlands	8	12	12	10	10
Yorkshire and the Humber	2	14	4	4	6
Southeast England	8	2	4	18	9
Southwest England	6	10	8	4	7
East of England	8	4	12	10	8
London	32	26	32	20	27
Scotland	10	2	4	4	5
Wales	2	2	0	4	2
Northern Ireland	2	0	0	0	1

PE = pulmonary embolism; PH = pulmonary hypertension.

**Table 2:** Routine investigation by respondents of the possibility of suspected CTEPD in patients following acute PE.

	Cardiology (*n* = 50)	Respiratory Medicine (*n* = 50)	Haematology (*n* = 25)	Internal Medicine (*n* = 50)	Overall (*N* = 175)
**Respondents who routinely investigate the possibility of suspected CTEPD in patients following acute PE, *n* (%)**	34 (68)	35 (70)	12 (48)	16 (32)	97 (55)
**Patients in which these physicians routinely investigate the possibility of suspected CTEPD**	*n* = 34	*n* = 35	*n* = 12	*n* = 16	*n* = 97
In all patients, including asymptomatic patients, *n* (%)	6 (18)	11 (31)	1 (8)	3 (19)	21 (22)
In patients who are symptomatic at the 3-month follow-up visit, *n* (%)	24 (71)	27 (77)	11 (92)	12 (75)	74 (76)
In patients with a large clot burden at acute presentation, *n* (%)	20 (59)	23 (66)	9 (75)	9 (56)	61 (63)
In thrombolysed patients, *n* (%)	8 (24)	20 (57)	7 (58)	6 (38)	41 (42)
In patients with thrombophilias, *n* (%)	12 (35)	14 (40)	1 (8)	5 (31)	32 (33)
In patients with recurrent VTE, *n* (%)	21 (62)	23 (66)	7 (58)	9 (56)	60 (62)
In patients with sPAP >60 mmHg at acute presentation, *n* (%)	14 (41)	18 (51)	5 (42)	8 (50)	45 (46)
Other, *n* (%)	1 (3)	2 (6)	0	0	3 (3)
**Tests used to investigate the possibility of CTEPD**	*n* = 34	*n* = 35	*n* = 12	*n* = 16	*n* = 97
**Echocardiogram (%)**					
Always/sometimes	91/9	83/9	83/17	56/38	81/14
Occasionally/never	0/0	9/0	0/0	6/0	4/0
**CTPA (%)**					
Always/sometimes	79/21	60/37	25/67	69/19	64/32
Occasionally/never	0/0	3/0	8/0	13/0	4/0
**V/Q or Q scan (%)**					
Always/sometimes	21/56	43/51	25/58	6/56	27/55
Occasionally/never	21/3	6/0	17/0	38/0	18/1
**CPET (%)**					
Always/sometimes	18/38	23/43	8/50	6/44	16/42
Occasionally/never	32/12	31/3	33/8	31/19	32/9
**6MWD (%)**					
Always/sometimes	26/38	23/46	17/50	13/50	22/44
Occasionally/never	21/15	26/6	33/0	19/19	24/10
**BNP or NT-proBNP (%)**					
Always/sometimes	53/35	34/37	17/58	25/38	37/39
Occasionally/never	6/6	14/14	25/0	31/6	15/8
**Lung function test (%)**					
Always/sometimes	47/35	49/34	50/33	25/44	44/36
Occasionally/never	15/3	11/6	17/0	25/6	15/4

6MWD = 6 min walk distance; BNP = brain natriuretic peptide; CPET = cardiopulmonary exercise test; CTEPD = chronic thromboembolic pulmonary disease; CTPA = computed tomography pulmonary angiogram; NT-proBNP = N-terminal pro-brain natriuretic peptide; PE = pulmonary embolism; Q = perfusion; sPAP = systolic pulmonary arterial pressure; V/Q = ventilation/perfusion; VTE = venous thromboembolism.

## Data Availability

The data presented in this study are available within the current article and its Supplementary Materials.

## Supplementary Materials

The following supporting information can be downloaded at: https://www.mdpi.com/article/10.3390/arm92010007/s1, Figure S1: Possible reasons for persistent dyspnoea following PE ranked in order of likelihood by respondents; Figure S2: Use of risk score calculators for estimating the probability of CTEPH by respondents; Table S1: General management of PE in the centres in which the respondents practice; Table S2: Respondents’ access to imaging modalities; Table S3: Experience in evaluating suspected CTEPD after PD by specialty; Table S4: Experience in evaluating suspected CTEPD after PE by region; Table S5: Criteria for referring patients to a specialist PH service in the centres in which the respondents practice; Table S6: Relative strengths and weaknesses of the imaging modalities in the context of PH.
