# Supplementary material for: Pulmonary Embolism (PE) to Chronic Thromboembolic Pulmonary Disease (CTEPD): Findings from a Survey of UK Physicians
Source: Adv Respir Med. 2024 Jan 9;92(1):45–57. doi: 10.3390/arm92010007 (PMC10801485; doi:10.3390/arm92010007)
Supplement: Supplementary file 1 [file arm-92-00007-s001.zip › arm-2686485-supplementary.pdf]

SUPPLEMENTARY MATERIALS

Table S1. General management of PE in the centres in which the respondents practice.

	Cardiology (n = 50)	Respiratory Medicine (n = 50)	Haematology (n = 25)	Internal Medicine (n = 50)	Overall (N = 175)
Local guidelines on PE management at the centre (work setting), n (%)					
Yes	(88)	(92)	(92)	(86)	(89)
No	(12)	(6)	(4)	(12)	(9)
Not sure	(0)	(2)	(4)	(2)	(2)
PERT at the centre (work setting), n (%)					
Yes	(40)	(54)	(48)	(14)	(38)
No	(54)	(40)	(36)	(70)	(52)
Not sure	(6)	(6)	(16)	(16)	(10)
Hospital runs a dedicated clinic for the follow-up of acute PE, n (%)†					
Yes, for all patients*	(44)	(66)	(52)	(42)	(51)
Yes, for hospital-admitted patients only	(22)	(10)	(16)	(10)	(14)
No	(26)	(22)	(24)	(36)	(27)
Not sure	(8)	(2)	(8)	(12)	(7)

* Including low-risk, outpatient-managed patients. † Of those managed as outpatients at the physician's centre.

PE = pulmonary embolism; PERT = pulmonary embolism response team.

Table S2. Respondents' access to imaging modalities.

	Cardiology<br>(n = 50)	Respiratory Medicine<br>(n = 50)	Haematology<br>(n = 25)	Internal Medicine<br>(n = 50)	Overall<br>(N = 175)
**Computed tomography, n**	**(n = 50)**	**(n = 50)**	**(n = 25)**	**(n = 50)**	**(n = 175)**
CTPA only	41	31	22	45	139
CTPA plus DECT	9	18	2	3	32
CTPA plus LSIM	3	11	1	1	16
Not sure which CT modalities, if any, are available	0	0	0	2	2
**Magnetic resonance imaging**	**(n = 41)**	**(n = 40)**	**(n = 21)**	**(n = 34)**	**(n = 136)**
Pulmonary angiography	26	30	14	20	90
Lung perfusion mapping	18	22	7	5	52
Not sure which MRI modalities, if any, are available	11	7	5	13	36
**Nuclear medicine**	**(n = 49)**	**(n = 47)**	**(n = 23)**	**(n = 48)**	**(n = 167)**
Q scan only	7	4	0	6	17
V/Q scanning	38	39	22	36	135
SPECT	14	15	3	6	38

Not sure which nuclear medicine modalities, if any, are available	1	2	1	5	9
**Other**	**(n = 15)**	**(n = 10)**	**(n = 4)**	**(n = 11)**	**(n = 40)**
Conventional pulmonary angiography/DSA	14	10	4	11	39
Other	2	0	0	0	2

CT = computed tomography; CTPA = computed tomography pulmonary angiography; DECT = dual-energy computed tomography; DSA = digital subtraction angiography; LSIM = lung subtraction iodine mapping; MRI = magnetic resonance imaging; Q = perfusion; SPECT = single-photon emission computed tomography; V/Q = ventilation/perfusion.

Table S3. Experience in evaluating suspected CTEPD after PE by specialty.

	Cardiologists (n = 50)	Respiratory Medicine (n = 50)	Haematologists (n = 25)	Internal Medicine Specialists (n = 50)	Overall (N = 175)
Do you know where your regional PH centre is to refer your CTEPD patients? (%)					
Yes	92	78	72	74	80
No	8	22	28	26	20
Do you consider your centre to have a protocol for the investigation of CTEPD following acute PE? (%)					
Yes	52	58	44	32	47
No	30	30	24	30	29
Not sure	18	12	32	38	24

CTEPD = chronic thromboembolic pulmonary disease; PE = pulmonary embolism; PH = pulmonary hypertension.

Table S4. Experience in evaluating suspected CTEPD after PE by region.

	Yes (%)	No (%)
Do you know where your regional PH centre is to refer your CTEPD patients? (%)		
Northeast (n = 7)	86	14
Northwest (n = 20)	80	20
East Midlands (n = 17)	59	41
West Midlands (n = 18)	89	11
Yorkshire and the Humber (n = 11)	100	0
Southeast England (n = 15)	53	47
Southwest (n = 12)	83	17
East of England (n = 14)	79	21
London (n = 47)	83	17
Scotland (n = 9)	89	11
Wales (n = 4)	100	0
Northern Ireland (n = 1)	100	0

CTEPD = chronic thromboembolic pulmonary disease; PE = pulmonary embolism; PH = pulmonary hypertension.

Table S5. Criteria for referring patients to a specialist PH service in the centres in which the respondents practice.

	**Cardiology (n = 50)**	**Respiratory Medicine (n = 50)**	**Haematology (n = 25)**	**Internal Medicine (n = 50)**	**Overall (N = 175)**
Persistent symptoms after 3 months of anticoagulation, before conducting further tests	44%	48%	32%	38%	42%
Persistent symptoms after a PE even if echocardiogram and other tests are normal	42%	38%	44%	30%	38%
Persistent symptoms and abnormal investigations, such as Q scan, CTPA and CPET, but with a normal echocardiogram	56%	66%	36%	40%	51%
If the echocardiogram shows PH or right heart dilatation	50%	78%	80%	62%	66%
Only patients without significant comorbid conditions	2%	10%	0%	4%	5%
I don’t refer the patient to a specialist PH service	6%	0%	4%	14%	6%

CPET = cardiopulmonary exercise test; CTPA = computed tomography pulmonary angiogram; PE = pulmonary embolism; PH = pulmonary hypertension; Q = perfusion.

Table S6. Relative strengths and weaknesses of imaging modalities in the context of PH

Variable	Chest Radiography	V/Q Scan	SPECT/CT V/Q	Single-Energy CT Angiography[#]	Dual-Energy CT Angiography	MRI	Pulmonary Angiography
**PH detection**	+	–	–	+	+	+	–
**Evaluation of anatomic compartments**							
Lung	+	–-	+	+++	+++	–	–
Cardiac chambers	+	–	–	++	++	+++	–
Pulmonary vessels	+	+	+	+++	++++	++	++
Mediastinum	–	–	–	+++	+++	+++	–
**Assessment of PH aetiology**	++	++	++	+++	++++	++	++
**General strengths**	Readily available	Screening for CTEPH; SPECT currently replacing planar V/Q	Combined evaluation of lung	Excellent evaluation of aetiologies of PH	Assessment of anatomy and lung perfusion (iodine	No radiation; excellent evaluation of cardiac function	Planning of endovascular

			parenchyma with lung perfusion		maps) in a single test	and pulmonary flow in one examination	treatment (PEA, BPA)
**Weaknesses**	Limited role in the assessment of aetiology	Need further imaging to assess the cause of PH; interpretive limitations in test patients with comorbid conditions	Lung assessment limited; needs more validation; radiation dose added with use of CT	Limited haemodynamic assessment; limited evaluation of distal pulmonary arteries (beyond subsegmental level)	Needs validation for all dual-energy CT technologies	Limited in the evaluation of lung parenchyma; not widely available; more technical expertise needed	Absence of perivascular structure evaluation; invasive test
**Average effective radiation exposure (mSv)**	0.05	2.2	2.6–3.5	2–5	3–5	None	10–30

Reproduced from Remy-Jardin et al. [18].

BPA = balloon pulmonary angioplasty; CTEPH = chronic thromboembolic pulmonary hypertension; PEA = pulmonary endarterectomy; PH = pulmonary hypertension; V/Q scan = ventilation–perfusion scintigraphy.

– = no utility, + = limited utility, ++ = moderately useful, +++ = useful, and ++++ = very useful. # Non-electrocardiogram-gated CT.

Figure S1. Possible reasons for persistent dyspnoea following PE ranked in order of likelihood by respondents.

Q. Please rank the following in order of which you would consider the most likely reason for persistent dyspnoea after 3 months of effective anticoagulation.

Ranking	Chronic pulmonary thromboembolism without PH (N=174)	CTEPH (N=175)	Lung disease (N=175)	Left heart disease (N=175)	Deconditioning (N=174)	Dysfunctional breathing/anxiety (N=175)	Other (N=106)
1	20%	32%	21%	5%	18%	4%	0%
2	25%	17%	21%	18%	10%	10%	0%
3	18%	13%	24%	22%	10%	13%	0%
4	16%	15%	19%	18%	13%	18%	0%
5	14%	11%	11%	17%	24%	22%	1%
6	6%	11%	3%	19%	23%	32%	8%
7	1%	1%	1%	0%	1%	1%	91%

CTEPH = chronic thromboembolic pulmonary hypertension; PE = pulmonary embolism; PH = pulmonary hypertension.

Figure S2. Use of risk score calculators for estimating probability of CTEPH by respondents.

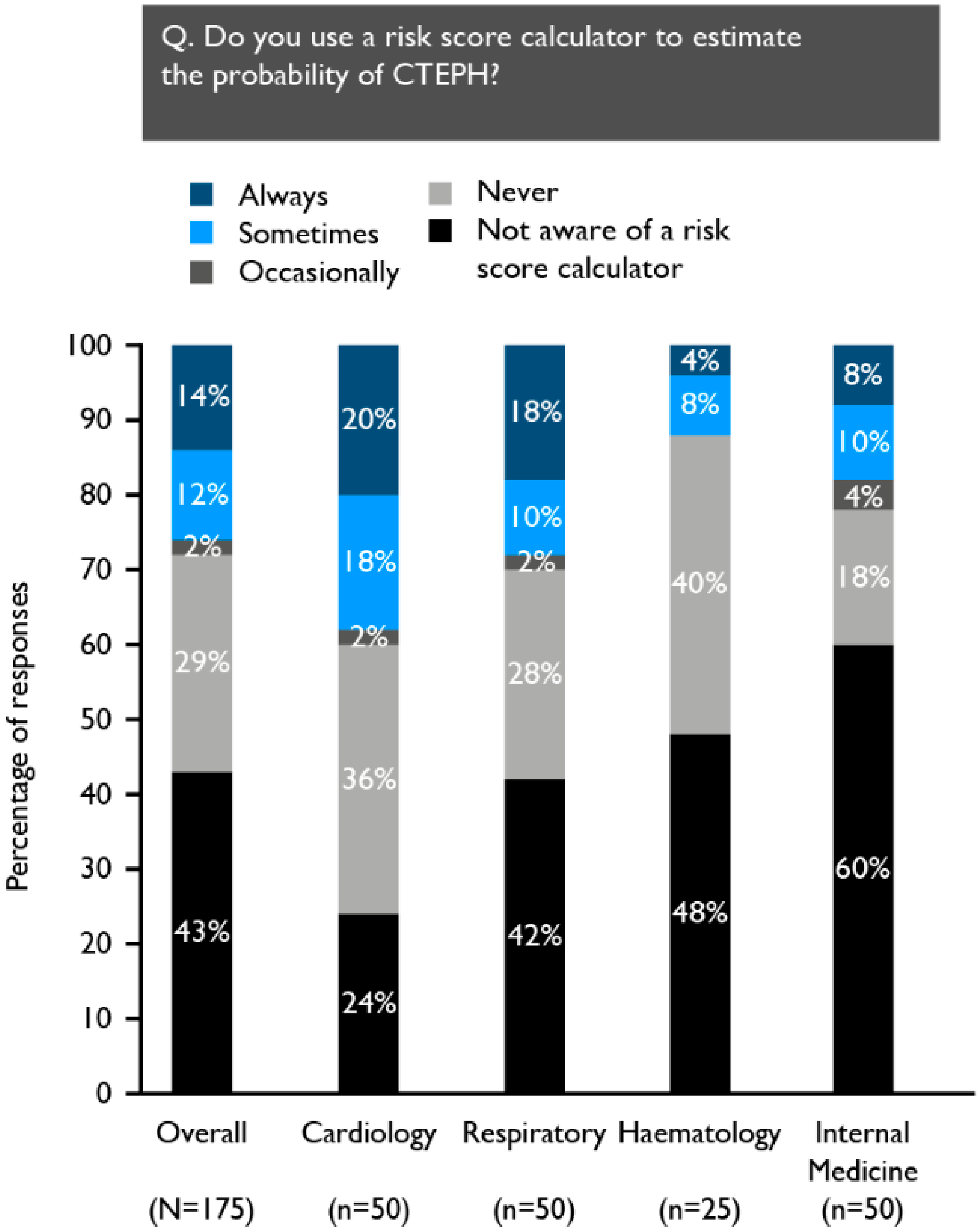

CTEPH = chronic thromboembolic pulmonary hypertension.
